# An intervention to stop smoking among patients suspected of TB - evaluation of an integrated approach

**DOI:** 10.1186/1471-2458-10-160

**Published:** 2010-03-25

**Authors:** Kamran Siddiqi, Amir Khan, Maqsood Ahmad, Shafiq-ur-Rehman

**Affiliations:** 1 Nuffield Centre for International Health and Development, Leeds Institute of Health Sciences, University of Leeds, Charles Thackrah Building, 101 Clarendon Road, Woodhouse, Leeds LS2 9LJ, UK; 2 Association for Social Development, House No. 12, Street 48, F-7/4, Islamabad, Pakistan

## Abstract

**Background:**

In many low- and middle-income countries, where tobacco use is common, tuberculosis is also a major problem. Tobacco use increases the risk of developing tuberculosis, secondary mortality, poor treatment compliance and relapses. In countries with TB epidemic, even a modest relative risk leads to a significant attributable risk. Treating tobacco dependence, therefore, is likely to have benefits for controlling tuberculosis in addition to reducing the non-communicable disease burden associated with smoking. In poorly resourced health systems which face a dual burden of disease secondary to tuberculosis and tobacco, an integrated approach to tackle tobacco dependence in TB control could be economically desirable. During TB screening, health professionals come across large numbers of patients with respiratory symptoms, a significant proportion of which are likely to be tobacco users. These clinical encounters, considered to be "teachable moments", provide a window of opportunity to offer treatment for tobacco dependence.

**Methods/Design:**

We aim to develop and trial a complex intervention to reduce tobacco dependence among TB suspects based on the WHO 'five steps to quit' model. This model relies on assessing personal motivation to quit tobacco use and uses it as the basis for assessing suitability for the different therapeutic options for tobacco dependence.

We will use the Medical Research Council framework approach for evaluating complex interventions to: (a) design an evidence-based treatment package (likely to consist of training materials for health professionals and education tools for patients); (b) pilot the package to determine the delivery modalities in TB programme (c) assess the incremental cost-effectiveness of the package compared to usual care using a cluster RCT design; (d) to determine barriers and drivers to the provision of treatment of tobacco dependence within TB programmes; and (e) support long term implementation. The main outcomes to assess the effectiveness would be point abstinence at 4 weeks and continuous abstinence up to 6 months.

**Discussion:**

This work will be carried out in Pakistan and is expected to have relevance for other low and middle income countries with high tobacco use and TB incidence. This will enhance our knowledge of the cost-effectiveness of treating tobacco dependence in patients suspected of TB.

**Trial Registration:**

Trial Registration Number: ISRCTN08829879

## Background

### Tobacco epidemic

Eight out of every ten smokers live in low- and middle-income countries[1]. In several of these countries, tobacco use is on the increase. Consequently, 70% of the projected mortality secondary to tobacco use is likely to be borne by low- and middle-income countries[1]. In addition, people die at an earlier age in such countries, causing the loss of 20-25 years of productive life[2]. Cost of cigarettes is relatively high in low- and middle-income countries compared to food and other essential commodities, resulting sometimes in their substitution. Therefore, tobacco use puts enormous burdens on countries' already ailing economies and contributes to existing impoverishment. The WHO Framework Convention on Tobacco Control (FCTC)[3] uses international law as a lever to establish policies to reduce both supply and demand for tobacco[4]. The key recommendations include offering help to people who want to quit tobacco use[5].

### Treating tobacco dependence

It has been suggested that at least 180 million deaths related to tobacco can be avoided by reducing current tobacco consumption by 50%. Surveys suggest that three out of four smokers wish to quit and one-third attempt to quit every year[6]. However, only 1-3% are successful if attempting on their own[7]. There is overwhelming evidence for the effectiveness and cost-effectiveness of a number of psychological and pharmacological treatments for tobacco dependence[8,9]. The evidence suggests that both behavioural and pharmacological therapies have the potential to double the quit rates among smokers and are highly cost-effective[10]. In low and middle-income countries, spontaneous quit rates are lower suggesting an even greater need for supporting people who wish to quit, and an opportunity to have a higher health benefit.

One of the several challenges in treating tobacco addiction in low- and middle-income countries is the lack of resources and governmental funding for the implementation of effective interventions. Many countries facing a tobacco epidemic are also confronting other challenges related to communicable diseases and nutrition-related disorders. Therefore, it may not be possible for countries with limited resources to shift priorities. However, existing programmes (such as TB control) can be utilised to extend their role to tobacco control with minimum investment. The WHO recommends integration of such tobacco cessation interventions into an overall policy on tobacco control and incorporation of these programmes into general health care[8].

### Tobacco dependence and tuberculosis

Smoking is closely related to tuberculosis (TB), another big killer. TB is a major public health issue in several countries where nicotine use has reached epidemic proportions. For example, 15% of all new TB cases in the world occur in China where nearly two thirds of men use nicotine[11]. Smoking prevalence is generally high among TB patients. In India, TB patients are three times as likely to be smokers than rest of the population[12].

A number of recent reviews have suggested that smoking is strongly associated with increased rates of both TB infection and TB disease [13-18]. Passive smoking is a particular risk factor especially in children. One study highlighted the high risk of acquiring TB infection among children who live with other adults who have had TB and were also exposed to second hand cigarette smoke[19]. Smoking leads to faster progression and poorer prognosis of tuberculosis[20]. In relation to TB outcomes, smokers are also less likely to adhere to TB treatment and more likely to relapse after successfully completing treatment according to some studies[14]. Smokers have also been shown to have a high risk of dying secondary to tobacco use[13,16]. Since the proportion of people using nicotine is very high in countries where TB is a major problem, even a modest relative risk amounts to considerable attributable risk of infection to the population[18]. In countries where TB is endemic and the vast majority of the population has been exposed to TB at some point in their life, tobacco use is likely to lead to progression or reactivation of TB infection. Patients, especially males, who are diagnosed with latent TB infection are less likely to adhere to their treatment and more likely to progress to full blown tuberculosis[21].

#### Treating tobacco dependence among TB patients and TB suspects

The association between TB and tobacco epidemic implies that addressing nicotine use in global TB control is likely to be beneficial for controlling TB as well as chronic diseases. It therefore makes sense that brief advice or any other available treatment for smoking cessation should be included in the management plan of TB patients. There are unmet opportunities for prevention and control of tobacco use among TB patients. During a typical course of treatment for TB, patients are in regular contact with health professionals for at least six months. Patients are considered to be more receptive to health education messages and willing to modify their health behaviour when they are ill[22]. Patients' contact with a health professional whilst they are being investigated for TB is considered as one of the "teachable moments" which is highly likely to trigger behaviour change[23]. Therefore, it makes sense to include advice and treatment on smoking cessation for all patients coming in contact with TB services and not just TB patients.

#### Research problem and justification

Before recommending TB programmes to incorporate identification and treatment of tobacco use, it is highly desirable and ethical to assess the feasibility of such an intervention. We found one such study conducted in Sudan that examined the feasibility of adding a simple cessation intervention to the general health care provision for TB patients in a poorly resourced health system[24]. The study found that the health professionals were willing and able to support a high proportion of TB patients (44%) in smoking cessation. There is no published research to our knowledge that has evaluated the cost-effectiveness of identifying and treating tobacco addiction among TB patients or TB suspects using a randomised controlled trial. Such research is highly desirable. It will assist programme managers and policy makers to see how such an integrated approach between the two programmes can be effectively and efficiently incorporated. We plan to conduct this study in Pakistan, a low income country with a high incidence of TB and a high prevalence of tobacco use[25]. Recent emphasis on both Tobacco and TB control provides a window of opportunity to evaluate innovative solutions for both public health problems and make these accessible to the population in Pakistan.

#### Research questions

1. What is the effect of a tobacco cessation intervention, based on WHO's 'five steps to quit' model, on patients' point and continuous abstinence from tobacco use? [Effect evaluation]

2. To what extent do the health professionals communicate risks of tobacco use and benefits of its cessation to their patients? What are their experiences and opinions about this strategy? [Process evaluation]

3. How do patients experience the intervention for tobacco addiction? [Ethical evaluation]

4. What is the incremental cost-effectiveness ratio of the intervention for tobacco addiction compared to usual care? [Economic evaluation]

### General Objectives

We aim to develop and trial a complex intervention to reduce tobacco dependence among TB suspects based on the WHO 'five steps to quit' model. Although this work will be carried out in Pakistan, we expect our findings will have relevance to tobacco control in other low and middle income countries.

### Specific Objectives

We will follow the Medical Research Council framework (see explanation in Methods section) for designing and evaluating complex interventions. Our specific objectives are:

1. To use qualitative methods to inform the development of an intervention based on WHO's 'five steps to quit' model (Phase I).

2. To develop and pilot this intervention which is likely to consist of: (a) an illustrative desk-guide (on patient assessment, education and treatment of tobacco dependence) and a training manual on this desk-guide for training of health professionals; and (b) leaflets for patients and others in the house containing information on the harmful effects of tobacco and advice on stopping its use, implying both cessation and prevention of tobacco use (Phase II).

3. To evaluate the incremental cost-effectiveness ratio of this intervention compared to usual care by estimating the proportion of people successfully quitting tobacco use (Phase III).

4. To determine barriers and drivers to the provision of treatment of tobacco dependence within TB programmes (qualitative component of Phase III).

5. To promote and support long term implementation of this intervention (Phase IV).

## Methods/Design

### Conceptual and theoretical framework

We propose to evaluate the cost-effectiveness of a multi-faceted intervention to control tobacco dependence, which by definition is a 'complex' intervention. Such interventions in general consist of several inter-connected elements which are all important for their functioning[26]. Therefore, it is difficult to specify an active ingredient in a complex intervention. Evaluation is generally difficult as complex interventions are not easy to define, develop, document, standardise and reproduce[27]. MRC has produced a framework which divides such evaluation into different phases that can be sequential or iterative. Each phase answers a specific question that leads to the next phase of the evaluation.

The objectives in our study align with different phases of this framework as follows:

1. Phase I will help us in identifying the likely behaviour change-elements in relation to tobacco use for the intervention especially the training package for professionals (desk-guide and training manual), and the information leaflet for patients.

2. Phase II will help in developing and piloting the intervention to allow us to refine and standardise it, test its feasibility in a resource-limited situation and test the feasibility of various (research and routine) data collection tools.

3. Phase III will consist of a cluster randomised trial to assess the effectiveness of the intervention, a costing study to assess its cost-effectiveness, and a subsequent qualitative study to identify barriers and facilitators in delivering the intervention.

4. Phase IV will identify key activities required to scale up the intervention.

### Objective 1

To use qualitative methods to inform the development of an intervention based on WHO's five steps to quit model (Phase I)

#### Key activities

From a health systems point of view, we would be interested in understanding the local health care context for delivering the intervention. This would involve issues like current working environment, staffing, and workload issues. We will also explore the possibilities of funding NRT and bupropion, their registration and addition to the essential drug list. We will do this through conducting a workshop with the Tobacco Control Programme and other stakeholders at the start.

We will use qualitative approaches to understand: (a) the extent to which health professionals particularly doctors currently convey risks of tobacco use and benefits of its cessation to their patients; (b) how they perceive their own role, knowledge, skills and confidence in conveying these messages; (c) how their own behaviour towards tobacco use is going to affect their attitude towards patients using tobacco and the advice they might offer; (d) which professional group would be most appropriate to provide the different elements of the package e.g. initial advice, motivation assessment, counselling, follow-up; and (e) what would be most useful form and content of training package and tools to support them in this process. Among patients, especially presenting to primary care with respiratory complaints, we are interested in understanding: (a) to what extent they currently understand the risk of tobacco use and benefits of its cessation; (b) in what form and detail they would like to receive such information from health professionals; (c) what kind of visual images will be evocative but also culturally appropriate if used in education materials; (d) what are their common misconceptions about tobacco use; and (e) what is their attitude towards smoking cessation aids including willingness to pay for NRT (f) do women and men respond differently to advice by physicians versus others, and by advisors based on gender

We will use focus groups to identify common themes as these allow participants to interact with each other and stimulate and generate themes which are sometimes not possible with interviews[28]. We are not only interested in "what they say they do" (their belief system) but also in "what they really do" (their behaviour). FGDs are particularly useful in this context as participants often challenge each other and therefore provide more in-depth understanding of inconsistencies between reported and actual behaviour. We are also interested in the social and cultural constraints that modify behaviour and are generally brought up in FGDs.

#### Settings

Focus groups will be conducted in health centres which will be participating in the subsequent phases of the study.

#### Participants

We will conduct two focus groups each from the following three categories of informants i.e. six focus groups in total. We will invite 6-8 participants for each focus group from the following:

1. Doctors

2. Nurses and health technicians (paramedical staff)

3. Patients (tobacco users) with respiratory symptoms (one with males and other with females)

#### Sampling

Purposive sampling will be used to understand the breadth of variation as well as the typical attitudes and behaviours among professionals and patients. We will try to have participants from similar social class and backgrounds to allow enrichment of the data with minimum censorship.

#### Data collection

FGD will be facilitated by the research officer who will use a FGD topic guide which will be piloted with a group of health professionals and patients in another health centre. FGD will be tape-recorded.

#### Data analysis

We will use a sequential focus groups and analysis process so that the later focus groups can be informed by and test themes emerging from the earlier focus group discussions. We will conduct an inductive analysis by indexing through generating codes and identifying themes from the narrative text. Analysis will be conducted in the original language to identify and link themes. All narratives will be analysed separately first, followed by comparing and linking themes generated during the analysis across narratives.

#### Outputs

1. A training package and tools (both format and content) which are context specific and are likely to ensure health professionals have the knowledge, skills, motivation and confidence to convey the risks of tobacco use and the benefits of cessation to patients.

2. Culturally appropriate messages and the form in which they are communicated to patients that is likely to challenge their misconceptions, answer common queries, provide the right depth of knowledge, their attitude towards smoking cessation aids including willingness to pay for them and motivation for them to modify their tobacco-related behaviour.

3. An understanding of health system constraints and drivers in delivering the intervention including possibilities of paying for NRT and bupropion

### Objective 2

To develop and pilot this intervention which is likely to consist of: (a) an illustrative desk-guide (on patient assessment, education and treatment of tobacco dependence) and a training manual on this desk-guide for training of health professionals; and (b) leaflets for patients and others in the house containing information on the harmful effects of tobacco and advice on stopping its use, implying both cessation and prevention of tobacco use (Phase II).

#### Key activities

These can be divided into development and pilot.

##### In the development

Two different processes will be used to develop products for health professionals (training module & desk-guide) and patients (illustrative education tool & leaflet) respectively. We will convene a "local working group" of national (Pakistani) experts in tobacco control, primary care, health systems and district health services management, the district TB programme coordinator and primary care physicians and paramedics. This group will use the nominal group technique (a face-to-face group process technique for gaining consensus)[29] to agree the form and contents of the training module and desk-guide. We will use a decision framework based on the usefulness and applicability of the interventions. We will commission a local professional media company to develop illustrative education tools and leaflet. We will make sure that the education tools are prepared to be appropriate for both urban and rural population acknowledging different patterns of tobacco use and literacy. If necessary, we will produce separate versions of such tools. The product specifications will be based on the outcomes of the focus groups. The final product will be reviewed and approved by the local working group.

With permission, we will also make use of any existing materials developed by other international agencies, research groups or countries (such as the UK) which can be adapted to the context defined in the previous phase. Once we have developed draft materials, we will get external review from other national and international experts in the field.

##### In the pilot

The intervention will be delivered in six health centres (three urban, three rural). A decision to include these centres in the main trial will be taken at the end of the pilot depending upon required alterations in the intervention. In these centres we will introduce new tobacco use registers/questionnaires including queries such as: (a) current tobacco use (yes or no); (b) amount of tobacco use; (c) whether the patient is willing to try to stop tobacco use (yes or no); and (d) whether the patient has been registered to receive "five steps to quit" intervention[30]. We will develop and use separate data collection tools for people registered to receive the intervention. This will include a patient card filled in for every tobacco user who is willing to try to quit and a register of all such patients[30]. This will help us in assessing the feasibility of all these modifications and additions. 25 consecutive attendees who consent to participate and meet the inclusion criteria will be recruited in each centre (n = 100) and offered the intervention. Training evaluation of health professionals, in-depth interviews with patients and health professionals, and observations of the health professionals delivering intervention, will be carried out to identify: (a) any structural or processes barriers to the implementation; (b) modifications required in the data collection tools and systems; and (c) any modifications required in the training package based on participant observation and training event evaluation; (d) any changes in the assigned roles of health professionals within the package; and (e) any modifications required in desk-guide, patient education tool and leaflets based on observation of care delivery and discussion with care providers.

We will also estimate the validity of the subjective assessment of abstinence by using a cotinine test (or CO test) on all participants at follow up during this pilot. Depending upon the validity, we will decide to either use cotinine test (or CO test) in the actual trial or just the subjective assessment as a proxy.

Ultimately, based on the results of this pilot, we will develop a final version of the various components of the intervention. We will also modify the process for delivering the intervention if necessary.

#### Outputs

1. A refined training module and an illustrative desk-guide on patient assessment, education and treatment of tobacco dependence for health professionals' use.

2. An illustrative education tool for patients

3. Leaflets for patients and others in the house containing information on the harmful effects of tobacco and advice on stopping its use which has been piloted

4. Additional leaflet for the patient's family that will include an explanation of the impact of second hand smoke on the patient and others

5. A revised and refined process for delivering the intervention in the study sites

6. Appropriate and revised data collection tools and systems.

#### The Intervention and its key components

FIVE STEPS TO QUIT - The Intervention Model: This is based on the evidence-based recommendations for treatment of tobacco addiction published by WHO in 2001[8]. The same approach is being advocated by IUATLD, National US guidelines and NICE in the UK[23,31]. This model relies on assessing personal motivation to quit tobacco use and uses it as the basis for assessing suitability for the different therapeutic options for tobacco dependence. Thus, the approach maximizes the efficient use of nicotine replacement therapy (NRT) and bupropion.

Key Components: We would like to develop components of the "Five Steps to Quit" intervention model on the basis of the following principles:

• Based on best available evidence and following an approach recommended by international agencies such as WHO and IUATLD[23].

• Can be delivered in primary care setting by non-specialist health care staff (doctors or other non-medical personnel) integrated in their routine healthcare provision.

The intervention model consists of five key steps

1. Asking about the status of nicotine use;

2. Advising about the benefits of stopping nicotine use;

3. Assessing the motivation to stop its use;

4. Assisting in stop attempts through various therapeutic options; and

5. Arranging follow-up

Examples of the specific components for each step of the intervention are given in Appendix 1; this is a general guide only and modifications will be made during the development of the intervention.

### Objective 3

To evaluate the incremental cost-effectiveness ratio of these interventions compared to usual care by estimating the proportion of people successfully quitting tobacco use (Phase III).

#### Hypothesis

For this part of the project, we hypothesize that the 'five steps to quit' intervention (with or without offering the therapeutic option) will be more effective and cost-effective in getting people who are suspected of tuberculosis to quit nicotine use and remain quitters at both 4 weeks and six months after the treatment compared to usual care and information leaflet.

#### Settings

The study will be conducted in health centres in the public sector which are designated TB diagnostic centres (as per WHO's definition) in rural and urban settings in two districts in Punjab, Pakistan. This has been the setting of a number of research activities carried out by the investigating team over the last 12 years mainly in tuberculosis. Diagnostic centres, in a typical district in Punjab, are generally:

• Rural health centres - rural settings

• Tehsil headquarter hospitals - urban settings

Centres are typically staffed by one to three doctors, nursing staff, laboratory technician, pharmacist and community health workers. They are also equipped with a basic microbiology laboratory capable of examining sputum for AFB. The outpatient departments are open to the population of the catchment area including referrals from Basic Health Units (a single doctor manned primary care health centre). Patients with suspected tuberculosis once seen are screened with two/three sputum examinations. If diagnosed with TB, they are registered and commenced on anti-tuberculosis therapy.

#### Study Design

**Figure 1:**
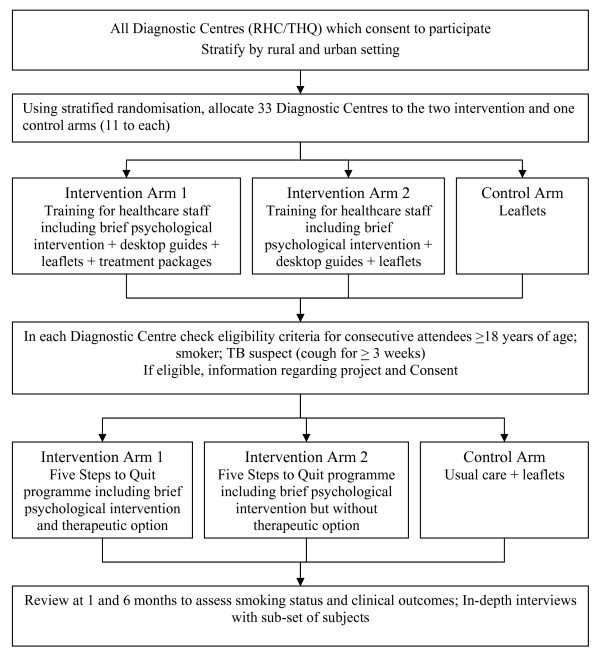
**Trial flow chart**.

To deliver 'five steps to quit' intervention (with or without offering the therapeutic option), individual patients cannot be randomised to the intervention and control arms within health centres due to the possibility of contamination. The most suitable design to assess its effectiveness is a cluster randomised controlled trial (RCT). The overall design is summarised in the trial flow chart in Figure 1. We propose to have three arms in the trial: (a) control arm - usual care and an information leaflet; (b) intervention arm 1 - 'five steps to quit' that includes offering therapeutic option; and (c) intervention arm 2 - 'five steps to quit' that excludes therapeutic option). We propose to select a total of 33 diagnostic centres (11 at each control and the two intervention arms) and to recruit 50 patients with suspected tuberculosis (see the section on sample size below).

#### Participants and Sampling

The sampling frame will consist of all TB diagnostic health centres that are approached for this study and their doctors and other paramedical staff have expressed willingness to participate. Access to these diagnostic centres will be facilitated by one of the investigating partner organisations, ASD, which has been given the responsibility of implementing the DOTS strategy in 18 districts in Punjab. Health centres in the sampling frame will be stratified according to rural (RHC) and urban location (THQ). 11 health centres will be randomly allocated to each of the two intervention arm and the control arms, using computer generated random number lists by an independent scientist.

In each health centre, we will approach adult patients (≥18 years), who cough for 3 or more weeks and are therefore suspected and screened for pulmonary tuberculosis. We would aim to recruit at least 50 patients from each centre over a period of three to six months. Patients will be provided with both verbal and written information about the study and only those who consent will be recruited in the study.

Patients whose condition requires hospital admission or other urgent medical attention will be excluded. Patients will be approached and recruited each day.

#### Delivery of the intervention

We propose to use a systematic, standardised approach to deliver 'five steps to quit' to make it effective and equitable[30]. It is envisaged that the intervention will be primary delivered by the TB DOTS facilitator based in the diagnostic centre with the help and under the supervision of the primary care doctor (Table 1). He will assess patients' eligibility for the study and send them to the TB DOTS facilitator for further assessment. All eligible patients will be provided with verbal and written information about the study and invited to participate. Patients who agree to take part will be taken through different components of the 'five steps to quit' programme in two appointments. Patients in intervention arm 1 will also be offered therapeutic option (Bupropion) and such patients will be referred to the primary care doctor for assessing suitability and prescribing Bupropion.

**Table 1:** Follow up and contacts with health professionals

Trial arms	First contact	FU at week 1	FU at week 5	FU at week 8	FU at week 25
Intervention 1 (brief psychological intervention + therapeutics)	Assess eligibility at the laboratory	TB DOTS facilitator	TB DOTS facilitator	TB DOTS facilitator	TB DOTS facilitator
	TB DOTS facilitator	Doctor	Doctor (if necessary)		
	Doctor				
Intervention 2 (brief psychological intervention only)	Recruitment at the laboratory	TB DOTS facilitator	TB DOTS facilitator		TB DOTS facilitator
	TB DOTS facilitator				
Control (usual care + leaflet)	Recruitment at the laboratory	TB DOTS facilitator	TB DOTS facilitator		TB DOTS facilitator
	TB DOTS facilitator				

The TB DOTS facilitators and primary care doctors will be given a training course (using training manual) on the "five steps to quit" programme on the use of desk-guide and focusing on[30]:

• The scientific basis of the intervention

• Techniques to enquire about the status of tobacco use

• Providing brief advice on the benefits of stopping its use through visual images

• Approaches to assess nicotine dependence and motivation to quit

• Using this motivation to propose various treatment options explaining their pros and cons

• Training in the use of available pharmacotherapies and other cognitive-behavioural strategies

• The procedure for follow-up and other technical and organisational aspects.

• Familiarity with the recording and reporting system such as revised records and new tools

Training of TB DOTS facilitators will also focus on the brief counselling techniques based on cognitive-behavioural model[32]. A simple desk-guide will be provided with simple algorithms and instructions for health professionals to follow "five steps to quit" programme. This will also include glossy images to support patients in understanding the benefits of stopping smoking. The TB DOTS facilitator, for all eligible patients, will be responsible for:

• Assessment of the status of their tobacco use through a checklist of questions

• Advising on benefits of quitting using visual and written educational material

• Assessment of willingness for inclusion in the study and motivation to quit using a simple scoring card

• Registration and recording of demographics and contact details

• Structured counselling using a desk guide and patient planner

• Giving a quit date after a week

On the subsequent visit in a week's time, TB DOTS facilitator will be responsible for

• assessing the status of their tobacco use

• Sharing their experience of attempting to quit, listen to the difficulties they had and providing supportive counselling to help cope with the difficulties

• Referring them to doctor if the intervention arm includes therapeutic option

• Arranging follow up in a month's time.

At follow ups at week 5 and 25, TB DOTS facilitators will be responsible for

• Reassessing status of nicotine use and recording it in the tobacco use questionnaire/register

• Reviewing treatment and other options

• Rearranging follow up as per desktop guide.

In the intervention arm 1, the primary care doctors, in addition to supervising TB DOTS facilitators, will be responsible for assessing and prescribing therapeutics (Bupropion) to patients who wish to quit and are being referred by the TB DOTS facilitators. All patients on treatment will have an additional follow up visit at week 8. In intervention arm 2, doctors will be only responsible for supervising TB DOTS facilitators. They will be provided with the appropriate training and relevant materials.

Apart from the relevant training and materials, health professionals will not be provided with any other incentive, financial or otherwise. However, treatments packs will be provided from the research budget. The research officer will also organise regular supervisory visits to oversee patient management and data collection procedures. Monthly cluster meetings of the health professionals involved will also be organised to discuss progress and potential problems.

#### The Control Arm

Participants in the control arm will also be recruited from those who are suspected of TB and screened using sputum examinations. The changes in the relevant registers will be made to record the status of nicotine use and health professionals will be given instructions on recording it on-site. The TB DOTS facilitators in control diagnostic centres will be provided with patient education leaflets to be given out to all patients recruited in the study. Apart from recording their status of nicotine use and arranging follow up at week 1 and 5, they will be provided usual care. No other components of the intervention will be delivered. We do not anticipate a significant risk of contamination, i.e. patients moving from one diagnostic centre to another, due to the geographical spread of facilities, and because no publicity will be produced regarding the availability of the treatment package in other facilities. In addition, we do not anticipate a significant benefit (other than what has been recorded previously in controls) in the control arm given that asking about nicotine use and providing information leaflets, are not, by themselves, sufficient to lead to stopping nicotine use.

#### Outcomes

These are as follows[30]:

1. Point abstinence at 4 weeks: The proportion of trial participants who have completely given up all forms of nicotine use at four weeks after the completion of bupropion and/or brief psychological intervention

2. Continuous abstinence up to 6 months: Proportion of trial participants who remained abstinent from 4 weeks onwards up to six months

3. We will also measure tobacco use e.g. number of cigarettes smoked per day to estimate any reduction in tobacco use secondary to the intervention

4. Secondary outcomes will include incidence of various adverse affects secondary to therapy

5. Economic outcomes will be assessed in terms of healthcare cost to get one person to stop smoking at four weeks. Healthcare cost will include the treatment cost, the average duration of health professionals' time spent with the patients during assessment, advice and counselling.

6. Process outcomes will include: the proportion of tobacco users who decide to quit and registered to receive 'five steps to quit' intervention; and the proportion of people registered who continue follow-up for the full period planned

#### Data collection

Our pilot study will determine the feasibility of our proposed tools to gather patient details and their status of nicotine use at various appointments. The modified tools will include:

1. 'Five steps to quit" intervention cards and register

2. A separate tobacco use questionnaire/register to record smoking status and motivation to quit

These approaches will allow us to record demographic details including gender, socio-economic status, presence of other smokers in the house and workplace, number of children at home, status of nicotine use, motivation level, type of therapy offered, extent to which therapy was used, adverse events and abstinence at different end points. We would adapt some of the tools already well described in the existing literature[30].

Patients who are subsequently diagnosed with TB will be followed up regularly for their TB treatment and the outcomes relevant to this study will be recorded during those visits. Trial participants who are diagnosed as not having TB and being given any of the three forms of therapy will be asked to follow up at the intervals specified. Controls not diagnosed with TB and received only usual care and a leaflet will be asked to return at 4 weeks to review their respiratory condition and to assess their status of nicotine use. Attrition is a potential problem. After piloting in phase 2, patients' mobile phone numbers will also be recorded and those who are lost to follow up will be contacted via their mobile phones to assess their status of nicotine use. A high proportion of Pakistanis possess mobile phones irrespective of socio-economic status.

Similarly, at six months in both intervention and control arms, all patients who stopped tobacco use will be contacted either via their mobile phones or landlines to assess their smoking status. According to our estimate this number will approximately be at least 264.

In order to estimate the average time spent by health professionals in delivering 'five steps to quit' intervention, our research officer will spent a day at each centre to measure and record this time.

We will also record the gender and status of nicotine use among all health professionals giving advice and include a column in the above patient record tools to record which health professional delivered which element of the package.

#### Data analysis

Our research officer will collate data from all health centres on a monthly basis and feed them into a database created via SPSS kept on a mobile computer. There will be an assessment of smoking status by intervention category, analysed on an intention to treat basis. The point abstinence will be compared between the intervention and control arms at 4 weeks after the completion of the treatment and in controls 4 weeks after they were given the leaflets. Further analyses will include the comparison of continuous abstinence at six months between the two arms. We will also estimate reduction in tobacco use in both trial arms and compare to detect any difference. Missing data on the outcomes will not be inputted. Secondary analyses will investigate the use of, and adherence with, the various forms of therapy. Further sub-analyses will be carried out to detect differences in outcomes according to a number of variables such as gender of the participants and providers, smoking status of the provider, socio-economic status, existence of other smokers in the house or workplace and presence of children at home. We are mindful that sample size estimation was not based on these sub-analyses and therefore we may not be able to detect small differences in the outcomes according to these variables.

#### Sample size

We have estimated a sample size of 33 clusters (diagnostic centres) 11 in each arm of the trial. Each cluster size will be 40. The actual number of patients recruited in each cluster will be 50 patients with suspected tuberculosis each [assuming 80% will complete the trial up to the review at 6 months as per study in Sudan[24]] to give 80% power to detect a difference in quit rates of 20% in the intervention arm compared with 10% in the control arm. The value of α is set at 0.05. The assumptions underlying this estimation are:

##### Quit rates

We base our cessation rates on the cumulative estimates in the recent systematic reviews conducted by Cochrane Tobacco Addiction Group [33-36]. According to these reviews, brief advice by physicians, nicotine replacement therapy (NRT), and use of bupropion can improve 6 month smoking quit rates to 74%, 77% and 94% respectively. All three approaches are part of our "5-steps to quit" intervention and it is reasonable to expect an effect size of at least 94% in our study. We would recruit our patients from a group who are suspected of tuberculosis with respiratory symptoms. It is likely that the quit rates may even be higher than the above in both treatment arms. The trial of treating tobacco addiction in TB patients in Sudan showed a sustained abstinence of 66% in the intervention group[24]. We also expect some loss of effectiveness due to the fact that this is not an efficacy trial and treatment will be provided integrated within an existing programme. However, we assume that this loss in effectiveness with the intervention strategy applied to routine practice will be off-set by higher quit rates in patients with respiratory symptoms suspected of tuberculosis. For the purpose of this study, we assume that the quit rates at 6 months would be at least 20% in the intervention group versus 10% in the control group.

##### Number of individuals per facility

On average 50 new TB patients are diagnosed every year in each diagnostic centre. The number of patients suspected of tuberculosis is generally ten times the number of patients diagnosed. Therefore, recruiting 50 patients from each centre is unlikely to pose any problems. In the study carried out in Sudan, researchers were able to recruit and enrol 44% of all TB patients[24]. We expect that on average one patient will be recruited each day. Recruitment will take place on five days of the week. After accounting for holidays and unanticipated recruitment difficulties, we expect to be able to recruit 50 participants with suspected TB from each facility within a period of three months.

##### Attrition Rates

The sample size calculation incorporates an attrition rate of 20%. This is based on the observations made in the trial of tobacco control in TB patients in Sudan where less than 20% patients defaulted over a period of six months. An intention to treat analysis will account for any non-differential loss to follow up. As part of DOTS, patients with diagnosed TB are generally followed up for a period of six months. However, follow up of non-TB patients will be arranged as part of the trial.

##### Intra-cluster variation

The intraclass cluster coefficient (ICC) of the outcomes is an important factor in estimation of the sample size for a cluster RCT. It is generally estimated from the previous studies of smoking prevalence and its outcomes in similar settings. However, we found very few studies reporting ICC in similar settings. We use an ICC of 0.036 which has been used estimated from trials conducted in primary care clusters in the UK[37].

### Objective 4

To determine barriers and drivers to the provision of treatment of tobacco dependence within TB programmes (qualitative component of Phase III)

#### Key activities

At this stage we will require an in-depth understanding of the way intervention would be implemented. We would like to know which factors would act as barriers to effective implementation and how health professionals would overcome these. We would also like to know the key drivers in the system which would be instrumental to successful implementation. We would be interested in understanding to what extent patient felt supported in their efforts to stop tobacco use through the health service intervention; which factors motivated them to quit; and which were their main constraints. Since this phase will be post-intervention, it would be preferable to use semi-structured interviews with key informants (see below) to gain this understanding.

#### Settings and Participants

We will recruit a group of study participants from the health centres from both urban and rural settings including:

• Patients who were offered intervention and did not quit tobacco use

• Patients who were offered intervention and quit tobacco use

• Doctors and programme managers

• Other paramedical staff.

#### Sampling

We will purposively select 16 individuals (8 from urban and 8 from rural settings) equally from all four key informant groups.

#### Data collection

We will use an interview topic guide which would be initially piloted with a patient and a doctor. All communications will be tape-recorded and transcribed. Key themes identified from an iterative analysis of focus group data from phase II will also be used to develop the interview topic guide.

#### Data analysis

We will use a sequential focus groups and analysis process so that the later focus groups can be informed by and test themes emerging from the earlier focus group discussions. Data will be categorised and coded for a thematic analysis. Initial codes, generated from the focus group in phase II will also be iteratively updated. Data will be organised to identify key themes. Dependability of these themes will be checked with respondents.

#### Outputs

1. Further refinement of the model of care and materials to take account of barriers and facilitators to implementation

2. Useful information to understand the implications of scaling up the intervention within TB programme

### Objective 5

To promote and support long term implementation of this intervention (Phase IV)

#### Key activities

Activities listed here will be ongoing throughout the study period. These include:

• Seek the national TB and Tobacco control programmes' endorsements for the intervention

• Work with the national programmes to develop a plan to scale up the intervention

• Develop a proposal to conduct embedded research to inform further scaling up.

## Discussion

### User participation

We will conduct patient focus groups in the first phase of the project to inform the development of the interventions, especially patient education tools. We will seek other opportunities to engage users in the research as appropriate e.g. we will endeavour to include patient representatives in the monthly cluster meetings. Although, we do not envisage reporting the results back to all research participants, we would convey our findings to the participating communities through local newspapers, radio, community leaders, patient representatives in the steering committees and posters in the participating health centres.

### Gender Considerations

We understand that gender is a key determinant of the attitude towards tobacco use and related behaviour. Gender also determines the different patterns and types of tobacco use. Furthermore, women's uptake of and response to NRT is different from men. The uptake of both men and women may also differ on the gender of the professional giving advice. Therefore, we will record gender of both providers and recipients and conduct sub-analyses to ascertain variable uptake, response to the treatment and maintenance of abstinence according to these gender differences. We will conduct separate focus groups of both women and men in phase I to capture gender perspective in attitudes towards tobacco use and its cessation.

### Confidentiality and data protection issues

The privacy of the participating patients will be protected, and all data will be coded and processed according to the rules and policies of University of Leeds.

### Ethical considerations

The effectiveness of specific intervention components (e.g. Bupropion) is established in trials conducted mainly in developed countries. However, the same is not true for the strategies that attempt to introduce these into existing health programmes. The proposed study is evaluating the effect of a multifaceted intervention to support patients who are suspected of TB to give up nicotine use. The research questions posed here respond to a priority need for developing countries where the existing health programmes provide an opportunity to control the increasing problem of tobacco addiction.

No participant will be deprived of any care that she would ordinarily receive. There will be no extra burden, financial or otherwise, on control subjects. Even participants in the control facilities may benefit from greater monitoring and attention. The four week follow up after their first contact is often required for their clinical condition. Upon completion of the trial, all control facilities will be offered a copy of all the training materials, training course, desk-guide and education tools. Under-powered trials are considered unethical[38]; therefore our sample size calculations are based on conservative estimates and high power.

Formal ethical approval is being granted by the Biomedical Ethics Committee of Pakistan Medical Research Council. An information sheet translated in Urdu will be given to all eligible patients. Patients will be given information on all aspects of the research project by means of verbal and written information at the inclusion. It will be clarified in the informed consent form that each patient can leave the study at any moment without having any repercussions on her usual care.

### Results and dissemination

Our aim is to ensure that the Pakistan National TB Programme and the Pakistan National Tobacco Control Programme adopts, scales up and sustains the intervention that we expect to arise from this work: that is, that the research benefits the people of Pakistan and not merely the researchers. History demonstrates that to achieve this aim, it is insufficient to follow the conventional approach of disseminating the research results at the end of the study.

Instead, our approach is to embed research within the national control programmes, starting even before the inception of the research and continuing beyond the end of the research to help the programme develop policy statements, implementation tools, training materials and courses. Policy-makers, researchers and other stakeholders are all involved throughout the process, from identification of priority problems, through broad development of research questions and proposals, regular feedback on progress of the research, interpretation of the research findings, and scale-up.

As a result, all stakeholders, including the Directors of the NTP and NTCP and the research team, have ownership of and responsibility for the research from start to finish. Thus research questions are asked which are of direct relevance to the control programme directors; candidate interventions are feasible in the economic, socio-cultural and political setting; and issues of scale-up are addressed at the inception of the research. This greatly enhances the likelihood that the research findings are incorporated not only into policy, but also into national practice. We will also make attempts to bring in other organizations, (e.g. NGOs, lobby groups working for tobacco control in Pakistan) to help with the dissemination of the results and use this research to lobby for greater tobacco control measures.

## Competing interests

We, as the authors of this article have no competing interests (financial or otherwise) in this publication.

## Authors' contributions

KS carried out the background literature review, identified research gap, developed research objectives and proposed research design. AK worked out the key components of the interventions and the mode of its delivery. MA developed detailed methods for the different phases of the study. SR developed detailed protocols for the delivery of intervention and data collection. All authors read and approved the final manuscript.

## Pre-publication history

The pre-publication history for this paper can be accessed here:

http://www.biomedcentral.com/1471-2458/10/160/prepub
